# Bronchoalveolar Results in Young Children with Chronic Lung Symptoms: Lessons Learned from an Allergy–Pulmonology Project Guided by an Updated Mini Review of the Current Literature of Bronchoalveolar Lavage Eosinophilia and Neutrophilia in Normal Children

**DOI:** 10.3390/children13020231

**Published:** 2026-02-06

**Authors:** Russell J. Hopp, Elizabeth A. Eischeid, Steven Rose, Heather Thomas

**Affiliations:** 1 Department of Pediatrics, Division of Allergy, Pulmonary and Sleep Medicine, University of Nebraska Medical Center, Omaha, NE 68198, USA; srose@childrensnebraska.org (S.R.); hthomas@childrensnebraska.org (H.T.); 2 Children’s Nebraska, Omaha, NE 68114, USA; 3 Department of Biology, College of Arts and Sciences, University of Nebraska at Omaha, Omaha, NE 68182, USA

**Keywords:** bronchoalveolar lavage, asthma, cough, bronchitis, pediatric

## Abstract

**Highlights:**

**What are the main findings of this study?**

- The background of BAL cellularity in normal children has not been updated for more than 25 years, which can serve as a reference point for comparing results in symptomatic children
- Bronchoalveolar lavage (BAL) in young children is usually done under circumstances of complex pulmonary symptomatology and comparing their results with normal children can be revealing.

**What are the implications of the main findings?**

- Eosinophilia is not expected in the lavage fluid of normal children, so its presence in a pulmonary symptomatic child should increase the likelihood of asthma consideration
- Elevated lavage neutrophils are not a finding in normal children, and their presence, when a majority percent of the total cells that exist, should increase the consideration for protracted bronchitis.

**Abstract:**

Background: Pediatric bronchoscopy and bronchoalveolar lavage (BAL) are valuable procedures, used by pediatric pulmonologists in a wide variety of clinical scenarios. Reports of indications for BAL include investigations of infectious processes, for unusual or poorly responsive pneumonia, and non-infections reasons, including interstitial lung disease and aspiration syndromes. BAL in pediatric asthma is occasionally done in severe and uncontrolled asthma, to rule out co-morbid conditions or to investigate asthma phenotypes. We report here the results of BAL in young children with global pre-BAL diagnoses, with comprehensive analysis of the BAL cellularity and culture results, and with a post hoc review by an allergist. The results of BAL in children with enigmatic pulmonary processes were compared to the expected BAL cellularity in normal children, obtained by an expanded historical mini review. Methods: The initial objective was to perform a mini review of the collective published data for normal/control children with BAL differential cell counts with the purpose of using the results to compare normals to the information obtained on the symptomatic children with BAL results from pulmonologists in our combined allergy–pulmonary division. The exploratory study group was children 0–6 years of age who underwent a BAL from 2000 to 2024 at an academic pulmonary-allergy division. The children had presumptive diagnoses requiring investigation, including the most common diagnoses of asthma, chronic cough, aspiration, or refractory bronchitis, and in this post hoc protocol only the diagnoses provided on the pre- and/or post-operative summary by the divisional pulmonologist(s) performing the BAL were used in the post hoc analysis. Secondarily, the operative day pre- and/or post-lavage diagnoses were used to divide the children into groups (based on operative day diagnoses) to stratify their lavage results, based on eosinophils, neutrophils, culture positivity and lipid-laden macrophages. Normative data collected from the literature was used as the historically expected results for the BAL group(s) analysis. Results: A mini review of BAL cellularity across 25+ years of literature was performed to establish normative data for our subsequent analysis. Both eosinophils and neutrophils are low or absent in normal children based on the comprehensive literature review. As a part of a larger cohort of 500 children ages 0–20 years, 317 children ages 0–6 were selected for review. The protocol was approved by the University Institutional Review Board. Using the mini review as reference, we found that eosinophil counts of one or more were recovered in over 20% of all children, regardless of bronchoscopy indication. Neutrophilia > 50% of cells and/or bacteria colony counts > 100,000 organisms were also frequent findings (>50 percent of the children). As a separate observation, lipid-laden macrophages did not isolate to aspiration indications for the bronchoscopy and lavage. Conclusions: An updated mini review of the cellularity expected in control children provided a context to the findings in our studied exploratory sample population. There was a high recovery of neutrophils coupled with culture positivity found across all children undergoing BAL. Eosinophils > 1 were present in up to 25% with a pre-lavage asthmatic symptom indication, but almost an equal percentage in other children with non-asthma-like conditions was surprising. Lipid-laden macrophage data was unhelpful.

## 1. Introduction

Bronchoalveolar lavage (BAL) findings are rarely in the purview of allergy immunologists, while pediatric pulmonologists perform the procedure on a routine basis. The information gained with cytology and bacteriologic analysis is valuable for current and future clinical care [1,2]. In a unique situation where allergy and pulmonary co-inhabit a divisional structure, the review of BAL results is a frequent conversation in clinical care conferences. An interesting historical perspective reviewing the utility of bronchoscopy in severe pediatric asthma is available [3].

Eosinophilia is considered a common denominator in both allergic and asthmatic states [4], and peripheral eosinophilia is considered a risk factor in the Modified Asthma Predictive Index (mAPI) [5]. The mAPI and the Pediatric Asthma Risk Score (PARS) use clinical data to predict asthma in young children, including peripheral eosinophilia [5,6].

Pulmonary function testing is not generally available until age four or older and forced impulse oscillometry is rarely available. Exhaled nitric oxide is often under-compensated, so it is often limited in clinical practices by financial constraints and is difficult for younger children to perform. Thus, children in the pre-school range often have minimal and/or age-limited objective data of asthma assessment. If, in a young child, objective BAL cellularity is available, its information may be the only objective data for clinical decision making.

Our mini review of BAL data in control children since 1994 showed that virtually no eosinophils and minimal neutrophils should be seen in the cellular differential, and by inference the bacteriological presence would be negative in those same control children (Table 1) [1,7,8,9,10,11,12,13,14,15,16,17,18,19,20,21,22,23,24,25,26]. The presence of an eosinophil(s) or exaggerated neutrophils provides definite clinical context as they are not expected as normal lavage resident cells [1,7,8,9,10,11,12,13,14,15,16,17,18,19,20,21,22,23,24,25,26].

As a sub-analysis of the student research project, we analyzed five years of BAL results from an academic pediatric medical center, using the lavage cellularity and bacteriological results in children six years and less. The results were reviewed by a pulmonologist and an allergist, to provide a unique multi-experiential dimensional perspective on the data. We present here the results and conclusions drawn from a collaborative study undertaken by the allergy and pulmonary academic division to compare the results of an exploratory sample of BAL cellularity in complex pulmonary-symptomatic children and their results compared to the results of our literature mini review of the expected cellularity findings in normal children. We sought to provide evidence that neither eosinophils or neutrophils are expected to be recovered cellular constituents in pediatric pulmonary lavage.

## 2. Methods

The mini review for establishing the expected reference differential BAL cell counts in normal (control) children was completed using Undermind (www.undermind.ai. 1111B South Governors Avenue, Suite 3611, Dover, DE 19904, USA), an AI-powered research assistant, followed by a Pub-Med cross-check, using the same search terms for both: pediatric, healthy, controls, and BAL cellular norms, focusing on English language as a priority. The discovered manuscripts were searched for references undiscovered by the two search strategies. The results were then used to compare the findings seen in a sample population of children in an academic medical center by a group of pulmonologists in an allergy–pulmonary division. The overriding premise of the protocol was that neither eosinophilia nor neutrophilia should be present in a lavage, and how the outcomes of the lavage data should trigger further investigation.

The protocol was initiated as part of a summer undergraduate research project sponsored by University of Nebraska Medical Center (UNMC) and Child Health Research Institute (CHRI) at Children’s Nebraska (1 June 2025 to 30 August 2025). The student (EE) was sponsored by an author (pulmonologist), and the project approved by the Institutional Review Board at UNMC # 309-25-EP, with an approval start date of 25 June 2025. The data was de-identified for name and address. Age was included for statistical analysis. Gender was not used in the analysis. Race and/or ethnicity was provided but not used in the analysis. The IRB allowed data extraction for the operative (BAL) day only based on a provided medical record number. The analysis supervisor (allergist) was blinded to all identifiers.

Since this post hoc protocol limited the investigators by the IRB to the information provided on the operative day diagnosis, there was no allowance for chart surveillance for asthma medication, antibiotics, or esophageal reflux medications. The protocol was limited to the day of lavage diagnosis, and both the pre- and/or post-lavage diagnoses. The lavage data was separately extracted and then stratified by the group operative day diagnoses.

The IRB allowed 500 pediatric patients with the inclusion dates of 2000–2024. The analysis in this report was limited to 0–6 years of age to focus on children who had a BAL for a multitude of medical concerns, including asthma, bronchitis, aspiration, chronic cough, or similar chronic issues. After the 500 children were provided by the medical records department from 1 January 2000 to the stop date of 31 December 2024, the students reviewed and extracted from the medical record the BAL operative day diagnosis(es), the age, lavage cellularity, Lipid Index, cultures and organism(s) recovered. The IRB excluded a detailed chart review for all other laboratory, radiological medication, and non-BAL pulmonary assessment (i.e., PFT). For this analysis 317 children, 0–6 years, are included. The BAL was performed by the Pediatric Pulmonary faculty at Children’s Nebraska. None of the subjects were co-managed by the allergist (RJH).

Briefly, the BAL procedure followed established guidelines [1]. The cytology, culture, and Lipid-Laden Macrophage (LLM) Index were performed using standard laboratory procedure.

## 3. Statistical Methods

Mean age and differences in age among the children were determined using *t*-test with *p* < 0.05 considered significant. Determination of differences in eosinophils, neutrophils, culture and lipid-laden macrophages was by a chi-square analysis with a *p* value < 0.05 considered significant.

## 4. Results

The last review of lavage cellularity had been a five-study report in 1995. To expand the database of normal/control children with lavage results we searched the discoverable results of normal/control children for BAL cytology after 1995 through the current literature. Table 1 shows the results of eighteen studies with reported cell counts, and five opinion publications which also referenced the discovered manuscripts and/or speculated on the likely recoverability of cells in lavage fluid. Based on the results of our mini review of existing studies we choose an eosinophil count of one or more as a relevant eosinophil number (count), and 50% or more of neutrophils (of the percent of 100 cells) as a conservative indicator of an inflammatory component to the lavage. Colony counts > 100,000 organisms were the usual cut-off for the pulmonologist doing the lavage at our institution and were kept as significant results. The mini review could also serve as a reference for all pediatric bronchoscopists as the data has multiple international sources.

For our exploratory sample three-hundred seventeen children were included from the larger population defined by the protocol. The BAL operative diagnosis could include two or more relevant pulmonary concerns (i.e., asthma and chronic cough), so the analysis investigated overlapping possibilities. The IRB limited our access to the chart for each subject enrolled, so the data extracted from the operative day report submitted by the pulmonologist was the only available result and limited us to a descriptive analysis of the information from the operative report diagnoses and the lavage data.

In the overall group, the mean age was 2.0 +/− 1.41 years. Racial/ethnic identity was collected but not utilized in the analysis. It was thought there was no influence of the ethnicity of the subjects in the analysis. Sixty-six percent of the children were male. There were no significant age differences between the groups defined by the BAL diagnoses (Table 2).

The BAL-day diagnostic likelihood(s) are presented in Table 2, along with the eosinophils (% per 100 cells) recovered in each group and sub-group. Group 1 had a pre-BAL diagnosis of asthma, while Group 2 were the remaining children without an asthma pre-diagnosis. Group 2 was then distributed into Groups 3–6 for further stratification (at least one of the diagnoses listed but not asthma) for an analysis. However, Group 6 had no asthma, and none of the other listed diagnoses.

Groups 3–5 could have more than one diagnosis, so the total n is >178 (no asthma) and is not discrete. If aspiration was a concern, the information for barium swallow or reflux medication was not permitted to be extracted but could be information known to the pulmonologist.

Using eosinophilia as critical element of cellularity of the lavage fluid, one or more eosinophils were seen in 28% of children with an asthma concern at BAL; meanwhile, in the non-asthmatic group and the different non-asthmatic groups a 20% recoverability of one eosinophil or more was found, but with a non-significant difference between Groups 2–6 and Group 1.

Table 3 defines the presence of percent neutrophils/per 100 cells counted in the lavage fluid. Group 1 was asthma, Group 2 the remaining children without asthma, and Groups 3–6 were the Group 2 children but further stratified and had at least one of the diagnoses listed, but not asthma. Group 6 had no asthma, and none of the other listed diagnoses. Approximately half of all BAL results revealed a 50% or greater recoverability of neutrophils.

Table 4 divides the groups into colony count ≥ 100,000 organisms with the neutrophil count 0–50%, or 50% or above (per 100 cells), or neither. There was not a significance of neutrophils tracking with a high bacterial count. Secondarily, the number of subjects with eosinophils ≥ 1 in the lavage was considered a potential variable of co-presence with neutrophils, as a potential cellular constituent that was present only based on the granulocyte load; however, eosinophilia ≥ 1 did not significantly track with elevated neutrophils (Data not shown). The groups were as previously defined.

Table 5 divides the lipid-laden macrophages (when reported) into 0–29 and 30 and above, with the aspiration subset (but no asthma) compared to the other non-aspiration groups. In fact, a LLM of 30 or higher was more commonly seen in the non-aspiration group.

## 5. Discussion

Bronchoalveolar lavage (BAL) is an available procedure for children with enigmatic respiratory problems, and the technical aspects and utilization concepts have been previously published [1,2,3]. Control children’s BAL cell count differential data for comparison is not limited, but recent reviews on normal or control children are less available, and our review extends the data through 2023. (Table 1) For comparison, a 1990 American Thoracic Society Steering Committee review of the BAL cellularity of nineteen studies with five to seventy-eight non-smoking adults reported that neutrophils ranged from 0 to 12% and eosinophils 0 to 1% [27].

To support a priori contention that eosinophils are not expected in BAL fluid in control children, and neutrophils should be of low numbers in the lavage cellularity, a mini review of published data in normal (control), healthy children was undertaken as basis for comparison of our exploratory population. The summary data (Table 1), the largest known compilation of normal/control pediatric BAL cytology, showed eosinophils are less than 1/100 cells counted, while resident neutrophils are less than 5/100 cells (Table 1). Thus, we established a cut-off of 0 vs. ≥1 eosinophils; although using 50 neutrophils/100 cells is 10× the expected, in this population it is a reasonable cut-off regardless of the lavage rationale (diagnosis), as neutrophilia was a common finding, and a cut-off needed to be established.

The second purpose of this report was to provide a report of the data obtained during a diagnostic BAL, comparing the operative report diagnosis to the objective results obtained at the BAL. An asthma possibility was not the only reason for the lavage but served as a convenient anchor point in the presentation of the 317 children with distinct reasons for their intervention. All children had complex pulmonary conditions, and the information obtained could be useful prospectively for the child receiving the lavage, but also as an object lesson in interpreting pediatric BAL information.

Our retrospective evaluation of BAL cytology in 317 children aged 0–6 years in our exploratory group who had underwent bronchoscopy for a variety of complex lung symptoms revealed a markedly different pattern of cellular presence than found in healthy children (Table 2). Macrophages were 1/3 of what is expected, largely replaced by neutrophils. Eosinophils were frequently recovered, even in children without a pre-procedure diagnosis of asthma. These findings coupled with culture results proved valuable for the pulmonologists performing the BAL and caring for the child. The post hoc comments by the allergy component of the project provides additional insights. It also suggests a pre-BAL checklist of the clinical status/medications (inhaled steroids) would be valuable, and that eosinophils should be reported on each cytology read-out as cells(s)/100 count.

Asthma, both allergic and non-allergic forms, is an eosinophil-associated disease [3]. Eosinophils are not always recovered from pediatric asthma BAL, but these children are often severe cases and are likely on asthma therapy for their disease [28]. Thus, eosinophils are expected but not universally recoverable in an asthmatic lavage. Much of the literature on young children with asthma symptoms is most often reported as having recurrent wheeze or chronic cough in published data [29]; meanwhile, in more recent BAL in children with asthma virtually all have severe disease and have potential medication interactions with cellularity findings (i.e., corticosteroids) [30]. Several publications in the pulmonary or allergy journals in the past 25 years reported lavage in more complicated asthmatic children. A study in 2002 of infants and children reported lavage eosinophilia in atopic older children, and scant eosinophilia in younger children [28]. Both groups had elevated neutrophils. A 1999 report of 14 asthmatics by Marguet et al. revealed eosinophils in some of the asthmatics [17]. An Italian study in 2010 by Snijders et al. reported on 91 asthmatics, with a range of eosinophils on 0–29% and range of neutrophils of 0–91% [31]. In summary it should be expected for a complicated pediatric asthmatic to have recoverable neutrophils (including excessive) and a large range of eosinophils, but zero eosinophils is not unexpected.

The presence of eosinophils in up to 28% of our studied children was the most critical finding from the allergist’s perspective. When eosinophil(s) (or basophils or mast cells) is reported in a BAL differential, the possibility of asthma should be strongly entertained, even if it had not been considered before the lavage. This would offer the opportunity, at follow-up, to perform appropriate asthma/allergy testing, when available, a CBC and differential (eosinophil count), and allergy skin testing and age-appropriate PFT. As an example, if a BAL is performed with a pre-school male (less likely a female) with a food allergy, or atopic dermatitis, and who subsequently has one or more eosinophils on a BAL differential, asthma is highly likely, and appropriate asthma therapy considered.

It is recognized that other uncommon pediatric lung processes can have recoverable lavage eosinophilia [32], including drug-induced, acute eosinophilic pneumonia, infant pulmonary eosinophilia (PIE), parasite-induced Churg–Strauss syndrome, and atypical chronic PIE. None of these diagnoses were included in the 317 children but that does not exclude an eventual diagnosis of any of these listed conditions.

The secondary finding of high neutrophil counts, >50/100 cells, was an expected result, based on the literature of BAL in children with lung disease in this age group [22]. Coupled with a high rate of colony counts ≥ 100,000 bacteria, the neutrophilia suggests a discovered protracted bacterial bronchitis, regardless of the pre-BAL diagnosis [33]. Even when bronchitis was a BAL concern, all other groups without a “bronchitis” indication also tracked with high neutrophils and/or high bacterial cell counts. The use of recent pre-BAL antibiotics was not tracked in our protocol (IRB exclusion) but should be considered on future pre-BAL checklists in clinical practice. BAL eosinophilia did not track with high neutrophil/bacteria colony counts (Table 3) which suggests a different chemoattractant mechanism for each granulocyte [34].

LLM levels > 30 were used as a marker of potential micro aspirations, but in our population the finding of high LLM was more discoverable in non-aspiration BAL diagnosis children [35]. The presence of a proton pump inhibitor or H_2_ receptor antagonist should also be included on a pre-BAL check list.

## 6. Conclusions

Guided by an updated mini review of cellularity results obtained during BAL in normal control children from 1987 to 2023, the BAL results from an academic pulmonary (and allergy) division for the years 2000–2024 were reviewed post hoc. The evidence of a very high frequency of neutrophilia and culture positivity, far beyond the normative data of the review, was confirmation of an excessive likelihood of protracted bacterial bronchitis, as reported in previous studies [33]. Furthermore, the recoverability of eosinophils was discovered in at least 20% of children undergoing the procedure. The presence of this biomarker should assist with potential asthma/atopy considerations for longitudinal care decisions. Lipid-laden macrophage data was not confirmatory of suspected micro aspiration. The information obtained during a BAL investigation can direct further post-BAL testing and therapeutic decision making. It also suggests that a checklist of recent/current medications would provide a framework for explaining the results of the lavage cellularity and efficiently improve post-lavage therapeutic and diagnostic considerations.

## Figures and Tables

**Table 1:** Previous BAL cellularity studies of asymptomatic (control) children.

First Author and Year	Macrophages	Lymphocytes	Eosinophils	Neutrophils	Other	Comment
Clement 1987 [7]	89.7	8.7	ND	1.3	N/A	N = 11, Control group 1–15 years
Ratjen 1994 [8]	81	16	0.4	1.9	N/A	N = 48, 3–5 years
Riedler 1995 [9]	91	7	0.2	3.5	N/A	N = 18, 0.3–10 years, Median data; pooled aliquots
Heaney 1996 [10]	70.8	3.8	0.4	5.7	mast cells and epithelial cells	UK, N = 55, through catheter in endobronchial tube
Tessler 1996 [11]	90	9	0	1.2		N = 16 controls, 1 month–16 years
Midulla 1995 [12]	87	8.7	0.2	5.5		Italian, N = 16, 1–36 months
Ratjen 1995 [13]	79	19	0	2	Stated as Granulocytes	N = 28, 3–16 years
Ratjen 1997 [14]	81	16	0.4	1.9	occasional basophil	German Language, English abstract N = 50, 3–15 years
Eber 1998 [15]						Children like adults
Grigg 1999 [16]	91.8	5.6	0	2.1		N = 12, Australia,
Marguet 1999 [17]	70	7	0	2		N = 10, 2.5–15 years
de Blic 2000 [1]						Summary of previous studies
Ahrens 1997 [18]	88.5	6.4	0.1	4.8		German, N = 21, 1–14 years
Koh 2007 [19]	85	7.5	0	2.3		Korean, N = 10, 1–4 years
Ferreria 2007 [20]	80	12	0.5	1.5		England, N = 5
Ratjen 2000 [21]						Summary of Previous studies [7,8,9,11,13]
Furtado de Mendonça Picinin 2010 [22]	80	8 to 16	<1	2		Opinion on cellularity. Portuguese [1,7,8,9,11,12,20]
I.F. de Mendonca-Picinin 2011 [23]	83	13.7	1	2	occasional basophil	Brazilian 1–8 years, n = 11
Meyer 2011 [24]	80–90	15-May	<1	<3		Opinion on cellularity
Gadaris 2010 [25]	93	3.93	0	2.94		Greek, N = 20, 1–14 years
Radhakrishan 2014 [2]						Referenced Meyer 2011 [24]
Pavic 2023 [26]	92	4	0.2	3.6		Croatia, N = 16, 1–16 years

**Table 2:** Determination of lavage eosinophilia in the BALday operative report diagnoses-defined groups.

Group	Diagnosis on BAL Surgical Report	N	Number with >1 Eosinophil on Cell Count and Percent (%)	*p* Values as Comparison for Eosinophil (s)
1	Asthma as a BAL concern	139	40 (28)	
2	No Asthma as a BAL concern	178	36 (20)	1 vs. 2 NS
3	Aspiration	108	23 (21)	1 vs. 3 NS
4	Chronic cough	33	7 (21)	1 vs. 4 NS
5	Bronchitis (subgroup of #2)	107	20 (19)	1 vs. 5 NS
6	Diagnosis other than Groups 3–5	38	8 (21)	1 vs. 6 NS

**Table 3:** Determination of lavage neutrophils in the diagnoses-defined groups.

	Diagnosis on BAL Surgical Report	N	Greater than 50% Neutrophils and Percent (%)	*p* Values as Comparison for Neutrophils
1	Asthma as a BAL concern	139	67 (48)	
2	No Asthma as a BAL concern	178	84 (47)	1 vs. 2 NS
3	Aspiration	108	45 (41)	1 vs. 3 NS
4	Chronic cough	33	17 (51)	1 vs. 4 NS
5	Bronchitis	107	51 (47)	1 vs. 5 NS
6	Diagnosis other than Groups of #3–5	38	21 (55)	1 vs. 6 NS

**Table 4:** Recovery of neutrophils and/or bacterial culture positivity in the groups based on diagnosis.

	Diagnosis on BAL Surgical Report	Neither Neutrophils > 50% or 100,000 Colony Count	Greater than 50% Neutrophils or >100,000 Colony Count	Greater than 50% Neutrophils and ➢ 100,000 Colony Count	*p* Value
1	Asthma as a BAL concern N = 138	36	55	52	
2	No Asthma N = 178	50	66	56	1 vs. 2 NS
3	Aspiration	27	43	35	1 vs. 3 NS
4	Chronic cough	7	15	9	1 vs. 4 NS
5	Aspiration	23	47	37	1 vs. 5 NS
6	Diagnosis other than Groups #3–5)	14	10	11	1 vs. 6 NS

**Table 5:** Lipid-laden macrophage positivity in aspiration vs. no aspiration diagnosis.

	Diagnosis on BAL Surgical Report	LLM 0–29	LLM 30 or Greater	*p* Value
1	Aspiration as a BAL day operative report concern	96	9	
2	No aspiration as a BAL day operative report concern	93	24	1 vs. 2 *p* = 0.014

## Data Availability

The original contributions presented in this study are included in the article. Further inquiries can be directed to the corresponding author.
